# HIV Transmission among Men Who Have Sex with Men due to Condom Failure

**DOI:** 10.1371/journal.pone.0107540

**Published:** 2014-09-11

**Authors:** Robert S. Remis, Michel Alary, Juan Liu, Rupert Kaul, Robert W. H. Palmer

**Affiliations:** 1 Ontario HIV Epidemiologic Monitoring Unit, Dalla Lana School of Public Health, University of Toronto, Toronto, Canada; 2 Centre de Recherche, CHU de Québec, Québec, Canada; 3 Département de Médecine Sociale et Préventive, Faculté de médecine, Université Laval, Québec, Canada; 4 Institut National de Santé Publique du Québec, Québec, Canada; 5 Department of Medicine, University Health Network, University of Toronto, Toronto, Canada; The University of New South Wales, Australia

## Abstract

**Background:**

Despite preventive efforts, HIV incidence remains high among men who have sex with men (MSM) in industrialized countries. Condoms are an important element in prevention but, given the high frequency of condom use and their imperfect effectiveness, a substantial number and proportion of HIV transmissions may occur despite condoms. We developed a model to examine this hypothesis.

**Methods:**

We used estimates of annual prevalent and incident HIV infections for MSM in Ontario. For HIV-negative men, we applied frequencies of sexual episodes and per-contact HIV transmission risks of receptive and insertive anal sex with and without a condom and oral sex without a condom. We factored in the proportion of HIV-infected partners receiving antiretroviral therapy and its impact in reducing transmissibility. We used Monte-Carlo simulation to determine the plausible range for the proportion of HIV transmissions for each sexual practice.

**Results:**

Among Ontario MSM in 2009, an estimated 92,963 HIV-negative men had 1,184,343 episodes of anal sex with a condom and 117,133 anal sex acts without a condom with an HIV-positive partner. Of the 693 new HIV infections, 51% were through anal sex with a condom, 33% anal sex without a condom and 16% oral sex. For anal sex with a condom, the 95% confidence limits were 17% and 77%.

**Conclusions:**

The proportion of HIV infections related to condom failure appears substantial and higher than previously thought. That 51% of transmissions occur despite condom use may be conservative (i.e. low) since we used a relatively high estimate (87.1%) for condom effectiveness. If condom effectiveness were closer to 70%, a value estimated from a recent CDC study, the number and proportion of HIV transmissions occurring despite condom use would be much higher. Therefore, while condom use should continue to be promoted and enhanced, this alone is unlikely to stem the tide of HIV infection among MSM.

## Introduction

The HIV epidemic among men who have sex with men (MSM) in Western industrialized countries began in the late 1970s. Several phases in HIV incidence have been observed since. From modeling and empirical studies in Canada, the US, and other countries, it appears that HIV incidence peaked at a high level in the early to mid-1980s and then dramatically decreased in the following decade [1]–[4]. This marked decrease in HIV incidence was due to the growing recognition and understanding of the modes of transmission of HIV infection and resulting reductions in risky sexual behaviours [5]–[6]. In particular, men who have sex with men reduced their number of sexual partners and began using condoms during anal sex on a large scale. Nevertheless, it appears that HIV incidence experienced a nadir (minimum) in the mid-1990s and subsequently increased thereafter. In Canada, HIV incidence increased by almost 70% among MSM from 1996 to 2006 [4], [7]. This increase was related to increases in risky sexual behaviour likely due at least in part to “treatment optimism” provided by the advent of highly effective antiretroviral therapy (ART) [8]–[9].

HIV incidence among MSM continues at high levels in most cities in Western industrialized countries. In fact, to the best of our knowledge, in no community has HIV incidence in MSM been significantly reduced in the previous 15 years. The persisting high HIV incidence in MSM may relate in part to the subpopulation of MSM who continue to engage in unprotected receptive anal sex. However, condoms are not 100% effective. A systematic meta-analysis of condom effectiveness in anal sex has not yet been carried out. With respect to vaginal sex, a Cochrane meta-analysis in 2002 observed an overall condom effectiveness of 80%, with a plausible range from 35% to 94% [10]. However, despite the fact that MSM are experienced in using condoms, condom use appears far from perfect. A recent study by D’Anna et al found a large proportion of cases where there was condom breakage and slippage as well as delayed application of condoms [11]. With respect to delayed application, i.e. penetration without a condom followed by the application of a condom before ejaculation, two studies observed an independent risk of HIV transmission among persons who have engaged in delayed application [12]–[13]. In addition to these factors, there may be leakage of virus-containing semen around the edge of the condom that is not perceived and is difficult to quantify [14]. Indeed, a recent study by Smith et al at the US Centers for Disease Control based on data from two large cohort studies in the US estimated that condoms were only 67% protective for HIV transmission through anal sex [15].

In light of the potential public health impact, we wished to examine whether condom failure might account for a significant proportion of ongoing HIV transmissions among MSM.

## Methods

In Ontario, since 1998, we have modeled annual HIV prevalence and incidence in each exposure category since the beginning of the epidemic. The present analysis was based on the latest estimate which was for calendar year 2009 [16]. In that year, we estimated that the HIV incidence rate among MSM in Ontario was 0.75% for a total of 693 incident infections. For the purpose of the present study, we calculated the likelihood of HIV transmission for each category of sexual exposure and fit the overall incidence to the modeled number of incident HIV infections. Sexual exposure by anal and oral sex were calculated; receptive and insertive anal sex were considered separately as was the use of ART in the HIV-infected partner, which is known to substantially reduce the risk of HIV sexual transmission. The model was programmed in SAS 9.3 (SAS Institute, Cary, NC) and Excel 2013 (Microsoft Office, Redmond, WA, USA).

### Population at risk and HIV prevalence

The number of males 18+ years of age and older in Ontario in 2009 was estimated to be 4,913,457 based on the census, as shown in Table 1 [17]. The number of MSM was derived using a triangulation methodology beginning with data from surveys of sexual orientation and refining the estimate to be consistent with data on HIV prevalence and HIV testing frequency in Ontario [16]. The prevalence of HIV was based on the Ontario modeling, taking into account the number of persons diagnosed with HIV, mortality among HIV-infected persons and the proportion of HIV-infected persons diagnosed. We estimated that 15,175 MSM in Ontario were infected with HIV as of end-2009, of whom 10,782 (71%) had been diagnosed. Further details of the methods and results of this modelling can be found in the 2009 Ontario HIV surveillance report [16].

**Table 1:** Model parameter values.

Variable description	Value
Ontario population	13,070,000
Number male	6,456,580
Number males 18+ years of age	4,913,457
Proportion of males 18+ who are MSM	2.2%
Number MSM	108,096
HIV prevalence	14%
Number HIV+	15,133
Proportion receiving ART	50%
Number receiving ART	7,567
Benefit of ART on reducing HIV transmission	96%
Number HIV–	92,963
Annual number of anal sex acts per person	100
Total number of anal sex acts among HIV–	9,296,256
Proportion anal sex with condom	91%
Proportion anal sex with a condom that are receptive	50%
Proportion anal sex without a condom that are receptive	40%
Annual number of oral sex acts per person	100
Total oral sex among HIV–	9,296,256
Per act risk of HIV infection:	
Receptive anal sex	0.0081
Insertive anal sex	0.00080
Receptive oral sex	0.00030
Insertive oral sex	0.000030
Condom effectiveness	87.1%

### Sexual practices, including condom use

Data on sexual behaviors were derived both from a literature review and from the Lambda study carried out in Toronto and Ottawa in Ontario in 2007 [18]. Values for frequency of sexual episodes in particular were guided by data from published studies [4]–[8], [19]–[21]. In the final model, the values for number of episodes of anal and oral sex were adjusted within the ranges of the values indicated in these sources such that the model generated the number of incident HIV infections from our estimates (i.e. 693 new HIV infections in MSM in 2009). The sources for these parameter values are described below and summarized in Table 2.

**Table 2:** Parameter values (base case, lower and upper 95% confidence intervals [CI]) for sensitivity analysis.

		Sensitivity analysis			
		95% CI			
Description	Base case	Lower	Upper	Distribution	Source
Proportion of males 18+ who are MSM	2.2%	1.8%	4.0%	Lognormal	[16]
HIV prevalence	14%	11%	17%	Normal	[16]
Proportion receiving ART	50%	40%	60%	Normal	[23]
Benefit of ART on reducing HIV transmission	96%	91%	99%	Normal	[30]
Annual number of anal sex acts per person	100	70	180	Lognormal	[4]–[9], [18]–[21]
Proportion anal sex with condom	91%	75%	96%	Lognormal (inverse)	[18]
Proportion anal sex with a condom that are receptive	50%	40%	60%	Normal	[18]
Proportion anal sex without a condom that are receptive	40%	30%	50%	Normal	[18]
Annual number of oral sex acts per person	100	70	130	Normal	[5], [18], [19], [21]
Per act HIV risk, receptive anal sex	0.0081	0.0050	0.0130	Lognormal	[25]–[27]
Per act HIV risk, insertive anal sex	0.00080	0.00050	0.00130	Lognormal	[25]–[27]
Per act HIV risk, receptive oral sex	0.00030	0.00010	0.00050	Lognormal	[25], [26], [29], [30]
Per act HIV risk, insertive oral sex	0.000030	0.000010	0.000050	Lognormal	[25], [26], [29], [30]
Condom effectiveness	87.1%	70.0%	95.0%	Lognormal (inverse)	[10], [11], [14], [15]

### Proportion of HIV-infected MSM receiving ART

The proportion of MSM receiving ART was derived from a database of diagnostic viral load testing in Ontario performed at the Public Health Ontario HIV Laboratory [22]. This was also reviewed in light of data from pharmaceutical manufacturers available indirectly which provided consistent results [23].

### HIV transmission probabilities

The probability of HIV transmission as a function of sexual practice was reviewed from modeling studies using empirical data to estimate the per-contact risk of HIV transmission independently for receptive and insertive anal sex [24]–[29]. There is general consensus that the HIV transmission rate associated with unprotected receptive anal sex is about 1.0%. For unprotected oral sex, we used 0.03% for receptive and 0.003% for insertive oral sex. It was assumed for the purpose of this study that the proportion of sexual acts with regular versus casual partners and the proportion of HIV+ MSM who had primary HIV infection and engaged in sexual behaviour in Ontario were not significantly different than the populations from which these transmission probabilities were drawn.

### Condom effectiveness

The estimate of condom effectiveness was based in large part on the results of a Cochrane meta-analysis as noted above [10]. We also examined studies reporting on rates of breakage and leakage of condoms used in anal sex [11], [14]. To avoid overestimating the role of condom failure in the final model, we used a higher value for condom effectiveness. However, a recent study from the US Centers for Disease Control estimated that condom effectiveness for preventing HIV transmission in anal sex was 67% [15]. The CDC study also found that intermittent condom use was not very effective and that not all MSM used condoms consistently.

### Impact of ART on transmission probability

Recent studies have found that the rate of HIV transmission in discordant couples was dramatically reduced in sexual partners of HIV-infected persons whose viral load was effectively suppressed by ART [30], [36]. We used a base estimate of 96% in our model.

### Fitting the model

The parameters, in particular, those related to frequency of sexual practices and condom effectiveness were adjusted to fit the number of HIV infections to the modeled annual estimate of incident HIV infections in 2009. The main objective of this study was to estimate the proportions of incident HIV infections that occur during anal sex with and without condoms and during oral sex. It was assumed that all oral sex occurred without condoms.

### Sensitivity analysis

We varied the parameters over a plausible range of values, with each model fitting the HIV incidence number to observed HIV incidence. The parameters which were subject to sensitivity analysis are shown in Table 2. This table presents the base case values of the parameters as well as the upper and lower plausible limits. All combinations of parameter values which yielded an estimated HIV incidence of 693 infections were combined to determine the variability in the proportions of infections due to each sexual practice. In addition, we also ran the model for two other values (one lower, one higher) of annual HIV incidence in Ontario.

## Results

The baseline values and the parameters underlying the model are presented in Table 1. Of the estimated 108,096 MSM in Ontario as of 2009, 14% were already HIV-infected, leaving 92,963 HIV-negative at potential risk for HIV infection. Each of these HIV-negative men had 9,296,256 episodes of anal sex and oral sex in 2009. The base case, plausible ranges, frequency distribution and data sources used in the sensitivity analysis are summarized in Table 2.

The final model incorporating the number of HIV infections in each category of sexual act (oral versus anal), stratified by whether contact was insertive or receptive, with or without a condom, whether or not the HIV-infected partner was receiving ART and the number of partners who are HIV-infected is presented in Table 3. HIV-negative men had 1,184,344 episodes of anal sex with a condom, 117,133 anal sex acts without a condom and 1,301,476 episodes of oral sex with an HIV-positive partner. The estimated number of HIV transmissions by sexual practice taking into account ART and condom use is shown in the second column from the right, namely 355, 227 and 112 infections, respectively. Of the 693 new HIV infections among Ontario MSM in 2009, 51% were related to transmission from anal sex with a condom, 33% through anal sex without a condom and 16% through oral sex.

**Table 3:** Number of sexual acts with HIV+ partners and HIV transmissions.

Transmission category	Number ofacts amongHIV–	Numberwith HIV+partners	HIV+partneron ART	Number ofepisodes	Preventiveimpact of ART	Per acttransmissionprobability	Condomeffectiveness	Number ofnew HIVinfections	Proportion ofnew infections
**Anal sex with condom**									
Receptive	4,229,796	592,172	ART	296,086	96%	0.810%	87.1%	12	
			no ART	296,086	-	0.810%	87.1%	310	
Insertive	4,229,796	592,172	ART	296,086	96%	0.080%	87.1%	1	
			no ART	296,086	-	0.080%	87.1%	31	
** Total**	**8,459,593**			**1,184,343**				**355**	**51%**
									
**Anal sex without condom**									
Receptive	334,665	46,853	ART	23,427	96%	0.810%	-	8	
			no ART	23,427	-	0.810%	-	190	
Insertive	501,998	70,280	ART	35,140	96%	0.080%	-	1	
			no ART	35,140	-	0.080%	-	28	
** Total**	**836,663**			**117,133**				**227**	**33%**
									
**Oral sex**									
Receptive	4,648,128	650,738	ART	325,369	96%	0.030%	-	4	
			no ART	325,369	-	0.030%	-	98	
Insertive	4,648,128	650,738	ART	325,369	96%	0.003%	-	0	
			no ART	325,369	-	0.003%	-	10	
** Total**	**9,296,256**			**1,301,476**				**112**	**16%**
									
**Total**				**2,602,952**				**693**	**100%**

**Figure 1:**
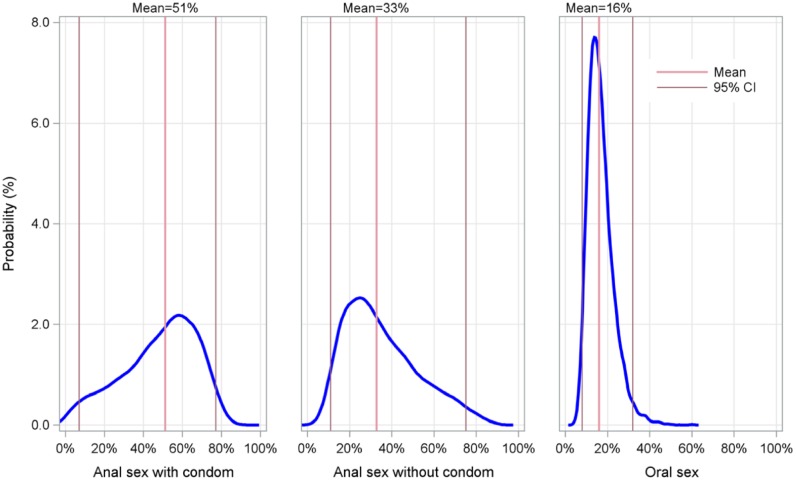
Modeled distribution of fraction of new infections due to each sexual practice, with mean and 95 percent confidence intervals.

Based on the sensitivity analysis, we observed considerable variability in the proportion of HIV infections among MSM due to anal sex with a condom, with 95% confidence limits of 7% and 77% (see Figure 1).

## Discussion

In a model of HIV transmission among MSM in Ontario, we found that the proportion of new infections related to condom failure was substantial and considerably higher than many may have previously thought. In particular, we found that 51% of new HIV infections in 2009 were related to condom failure during anal sex. This estimate of the proportion of transmissions occurring despite condom use is likely conservative (i.e. low) since we used a relatively high estimate (87.3%) for the base case value of condom effectiveness. However, a recent study from the US Centers for Disease Control estimated that condom effectiveness for preventing HIV transmission in anal sex was 67%, that intermittent condom use was not very effective, and that not all MSM used condoms consistently [15]. If condom effectiveness were closer to the value observed in this study, the proportion of transmissions occurring despite condom use would be substantially higher.

Condom failure is often due to breakage and slippage, sometimes, but not necessarily, due to non-optimal use [11], [14]. Delayed application where the condom is applied some time after penetration but prior to ejaculation has also been associated with transmission during anal sex [12]–[13]. Finally, condom failure may not always be apparent: HIV-containing semen may also leak around the condom edge.

Though our results may seem surprising, they are actually quite intuitive. If condoms are used in a majority of sexual acts and condom effectiveness is less than 100%, it follows logically that a significant proportion of HIV infections would be due to condom failure. This is analogous to the situation in vaccine-preventable infections in highly vaccinated populations when most persons who become infected have been vaccinated. For example, in a recent outbreak of mumps in New York City, 91.5% of cases had received at least one dose of vaccine and 74.2% of cases had received two doses [31].

Though our results are not mathematically surprising, it may mean that preventive messages historically disseminated to men who have sex with men should be modified. The prevailing message is that condoms reduce the risk of HIV and that, furthermore, the consistent use of condoms provides excellent protection against HIV. Nonetheless, while this message may need to be qualified, this is not to say that condoms have not played a significant role in decreasing HIV transmission rates among MSM, particularly compared to what might have occurred had this measure not been introduced and disseminated. While it is impossible to assess the hypothetical trajectory of the epidemic without the availability and widespread use of condoms for prevention, clearly it would have been significantly more severe. Thus, condoms have been and should remain an important tool in our armamentarium for reducing the risk of HIV transmission among MSM.

It may appear that the proportion of HIV transmissions due to oral sex is higher than would be expected, given the very low per-act risk of HIV transmission through either insertive or receptive oral sex [25], [26], [29], [30]. However, despite the low HIV transmission risk per oral contact, many MSM have increased the frequency of oral sex relative to anal sex because of the much lower rate of HIV transmission [11], [12], [32]. Therefore, we believe that the substantial relative increase in oral vs. anal sex (particularly unprotected) may explain the residual rate of HIV transmission through oral sex.

Although effective antiretroviral therapy dramatically reduces HIV transmission risk from an infected individual [30], [36], it is also clear that HIV transmission continues to occur in MSM populations in most Western industrialized countries virtually unabated over the past 15 years. Indeed, in most MSM populations, HIV incidence appears to have increased since the advent of highly effective ART in the mid-1990s. For example, in Canada, estimated HIV incidence in MSM in 2011 was 70% higher than in 1996 [1]. Therefore, although both condoms and ART have played a critical role in reducing HIV transmission among MSM, they have clearly not succeeded in controlling the epidemic in this population.

There is a potential risk in oversimplifying the dissemination of our findings, disseminating a message that condoms are not as effective as we might think. This could lead to a reduction in condom use and resulting increases in HIV incidence. This is not the intent of the present work, and nor is it the necessary implication of our findings. Rather, our results mean that condoms need to be used more effectively in this population, such that condom effectiveness can more closely approximate condom efficacy. Thus, we must not abandon our efforts to improve the best practice in terms of the use of condoms addressing the potential errors that may result in reduced condom effectiveness including such issues as placing the condom on the penis before any sexual contact. There is evidence from Ontario, for example, that some men are applying condoms partway through the sexual act and exposing their partners to virus that may be present and perhaps in high concentration in pre-ejaculate [12]. We did not assess the specific role of delayed application of condoms in the present analysis but this could well be a factor in ongoing HIV transmission. Rather than suggesting that condoms be abandoned since they are not fully effective, their use should be encouraged and reinforced to ensure that they are used to maximize their effectiveness in preventing HIV transmission.

Our results force us to consider whether condom use, either alone or in conjunction with high community rates of ART, is enough to control HIV transmission in men who have sex with men. For example, the use of pre-exposure prophylaxis among the most at-risk MSM could contribute to reducing HIV transmission in this population [33]–[35]. In addition, modifying other aspects of patterns of sexual behaviour may also be necessary. This could include reducing the number of sexual partners and selecting partners who are less likely to be HIV-infected which may in turn be related to where they are recruited (e.g. bathhouses) and type of partner (i.e. regular versus casual). This has historically been a sensitive issue but it is clear that the prevalence of HIV in sexual partners will determine the likelihood of HIV acquisition and the number of partners will also increase the chance an individual will have sex with somebody who is infected and become HIV-infected.
